# Wearable Sensors for Measuring Movement in Short Sessions of Mindfulness Sitting Meditation: A Pilot Study

**DOI:** 10.1155/2018/7275049

**Published:** 2018-05-07

**Authors:** Victor H. Rodriguez, Carlos T. Medrano, Inmaculada Plaza

**Affiliations:** ^1^EduQTech, EUPT, Universidad de Zaragoza, c/Atarazana 2, 44003 Teruel, Spain; ^2^IIS Aragón, Universidad de Zaragoza, Zaragoza, Spain

## Abstract

Mindfulness techniques are useful tools in health and well-being. To improve and facilitate formal training, beginners need to know if they are in a stable sitting posture and if they can hold it. Previous monitoring studies did not consider stability during sitting meditation or were specific for longer traditional practices. In this paper, we have extended and adapted previous studies to modern mindfulness practices and posed two questions: (a) Which is the best meditation seat for short sessions? In this way, the applications of stability measures are expanded to meditation activities, in which the sitting posture favors stability, and (b) Which is the most sensitive location of an accelerometer to measure body motion during short meditation sessions? A pilot study involving 31 volunteers was conducted using inertial sensors. The results suggest that thumb, head, or infraclavicular locations can be chosen to measure stability despite the habitual lumbar or sacral region found in the literature. Another important finding of this study is that zafus, chairs, and meditation benches are suitable for short meditation sessions in a sitting posture, although the zafu seems to allow for fewer postural changes. This finding opens new opportunities to design very simple and comfortable measuring systems.

## 1. Introduction

Around 1980, Jon Kabat-Zinn adapted some meditation skills originally from oriental traditions, provided them with a lay and scientific character, and applied them to reducing stress [1]. Kabat-Zinn defined mindfulness as the awareness that emerges through paying attention on purpose, in the present moment, and nonjudgmentally to the unfolding of experience moment by moment [2]. Since then, mindfulness has been increasingly applied in health and well-being [3–6].

Although there are two broad schools of mindfulness (a traditional Buddhist approach and a contemporary western approach), embodiment is a common process that can integrate them [7]. For mindfulness practice, the experience of being embodied involves paying attention to the body [8]. The observing part of body mindfulness is related to body awareness, defined as “the subjective, phenomenological aspect of proprioception and interception that enters conscious awareness” [9]. Body awareness is a key process in understanding the differences between meditators and nonmeditators [10].

Mindfulness manuals tend to recommend beginning with the daily practice of mindfulness of breath during formal seated meditation sessions [11]. Although some techniques recommend daily practice of 45–60 minutes, new studies recommend shorter sessions, between 5 and 15 minutes in the beginning stages [12]. Buddhist tradition texts describe the proper sitting posture: the back should be kept straight and the legs crossed. Similarly, sitting meditation is a basic technique in western mindfulness meditation [13]. A relevant aspect of the meditation posture is stability, and this can be achieved sitting in an upright posture that embodies dignity [12, 14]. Although a relaxed posture is adopted, prolonged motionlessness can lead to pain in muscles and joints. Mindfulness meditation instructors often encourage students not to shift position to relieve the pain but instead to focus careful attention directly on the pain sensations and to assume a nonjudgmental attitude toward these sensations to reduce the distress [3]. Recently, Jones [15] investigated the relationship between the posture and outcomes of mindfulness practice. Participants rated how comfortable they found each posture along with the difficulty to maintain it. This pilot study concluded tentatively that distress tolerance seemed to decrease in the slouched posture. Qualitatively, it appeared that the upright posture may facilitate breathing during mindfulness practice. Researchers could widen their focus to examine the role of the body in bringing about the beneficial outcomes of mindfulness-based interventions [15].

There are several kinds of meditation seats. Oriental meditation is performed while seated on a cushion in either the full-lotus or half-lotus position [16]. Instead of a cushion (zafu), a comfortable chair can be used for people with back pain or difficulty getting up [17]. Researchers have also used a kneeling meditation bench that is especially helpful for beginners to optimize spinal alignment and reduce weight and stress on the knees, hips, ankles, and back [18].

Monitoring the mindfulness practice is not a novel idea, and several studies measure physiological variables during meditation. Ahani et al. [19] recorded electroencephalographic (EEG) and respiration signals during a mindfulness meditation intervention. Arch and Craske [20] followed a focused breathing induction in which they controlled the heart rate (beats per minute). In a similar way, Vidyarthi and Riecke used two breathing sensors, which measure thoracic/abdominal expansion. Custom algorithms were created to extract parameters from the data: respiratory depth, respiratory length, and thoracic-to-abdominal ratio [21]. However, few of the previous studies considered stability or posture. In this regard, accelerometers have long been used to measure the posture [22, 23] or stability [24–27]. In the clinical context, stability has been referred to as a person's ability to maintain the position of the body or, more specifically, its center of mass, within specific boundaries [28]. For example, stability of construction workers [26], older people [27], or people with Parkinson's disease [25] has been measured, generally under conditions that challenge stability (stance on foam with eyes closed or posture with loads). In the present study, stability is understood in a more general perspective, and it is considered to be equivalent to lack of motion. Ideally, the meditation posture favors stability. Chang et al. were pioneers [29, 30] in this topic and proposed measuring vibration degrees as a good index in training body stillness. A lower motion would mean a better meditation state. These authors propose a body stillness monitoring system based on a triaxial accelerometer. Both a mean motion index and a maximum motion index were derived from the square summation of three axes. In a posterior study [31], they used the mean motion index that was extracted from accelerometers placed on the arm and on the chest in controlled wide amplitude movement experiments. It was determined that the arm was a more sensitive location. Then, the sensor on the arm was used during Chan Ding practices. The mean motion index was shown to be different for inexperienced people.

Chang et al. [30] secured the measurement system using a belt between the abdomen and chest or on the arm [31]. Nevertheless, other authors describe other body locations (head, sternum, lumbar or sacral region, wrist, etc.) to measure postural sway, stability or balance, especially while standing [32, 33]. A review of commercial products in the related problem of postural control indicates the same variety of body positions for the wearable sensor: collarbone, the lower back, the back of the neck, or glasses [34–37].

The current pilot study is motivated by the possibility of measuring motion and postural changes in mindfulness sitting meditation. This would allow users to receive feedback on their progress and to facilitate further investigations about the role of movement and posture in the outcomes of mindfulness-based interventions. We will focus on two factors: the meditation seat and the sensor location. The contributions of this paper come from the answer to the following two research questions: (i) Which is the seat that most favors stability? In this way, we are expanding the range of applications of stability measures to meditation activities, in which the sitting posture should favor stability and low range of movements, and (ii) Which is the most sensitive location for inertial sensors to measure body motion? Our study differs from the work of Chang et al. [30, 31] in the characteristics of the meditation (beginner-oriented: only 10 min; no full-lotus or half-lotus required), in the inclusion of more sensor locations and the analysis of the influence of the seat.

## 2. Methods

### 2.1. Participants

The sample consisted of 31 participants, 16 males and 15 females, with a mean age of 28.8 (range 18–46). Out of the participants, 27 had no experience and four had previous experience in mindfulness meditation ranging from 4 to 20 years.

The study protocol was approved by the Committee of Ethics for the Clinical Investigation of Aragon (CEICA). All the subjects received the information about the study in oral and written format, and they approved it.

### 2.2. Procedure

**Figure 1:**
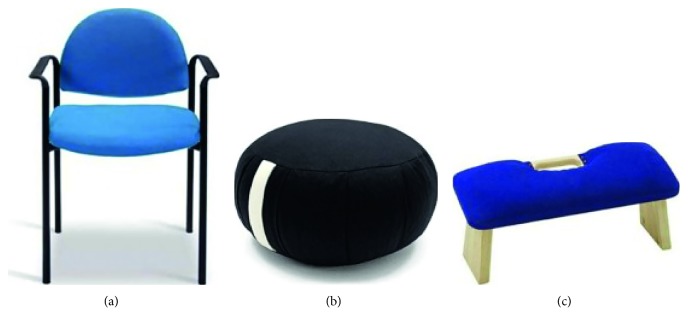
Meditation seats used in the short meditation sessions: (a) chair; (b) zafu; (c) meditation bench.

According to the literature review performed at the beginning of this work, several seats for sitting during mindfulness meditation were tested: a chair, a zafu (a small cushion), and a meditation bench (Figure 1).

The subjects were required to perform three times a short breathing meditation session, one for each type of seat. The order of the seats was randomly assigned but compensated so that the three seats were approximately selected equally for the first, second, and third sessions. Nobody chose a lotus posture (full or half) with the zafu. Each session lasted 10 minutes. The participants relaxed and rested 2 minutes between sessions (or longer in case of pain). Most mindfulness practices began with the observation of breathing [38], so the volunteers were asked to focus on their breath and to count groups of five breathing cycles (inhalation-exhalation). Whenever their attention wandered from their breath to inevitable thoughts and feelings that could arise, the subjects simply had to take notice of them and then let them go as attention was returned to breathing, using their breath as an anchor [13].

**Figure 2:**
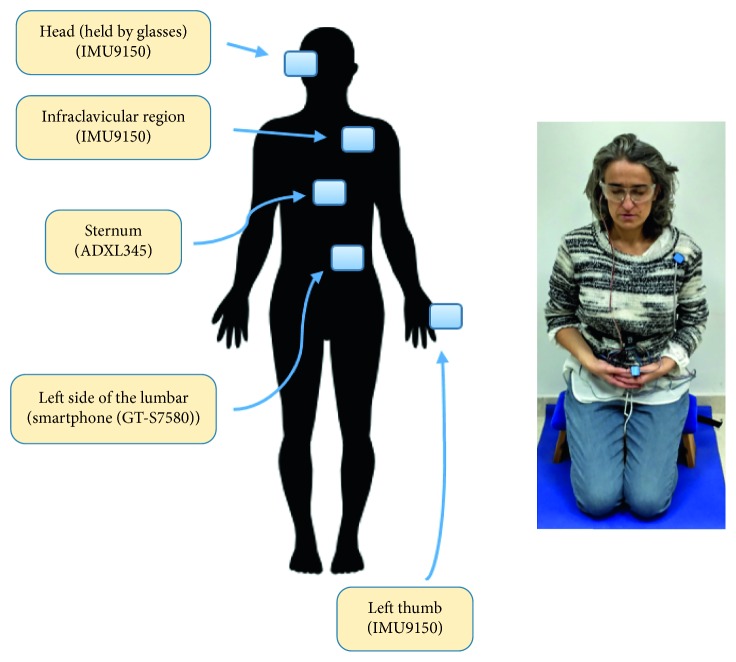
Schematic location of sensors on the body.

During the meditation sessions, a series of inertial sensors were placed on different body locations as described in Figure 2.

More specifically, the sensors were placed as follows:

1. An IMU9150 [39] (±4G, 16-bit resolution) on the hand, specifically, on the left thumb fastened with strips (from now on, this location will be referred to in short as *thumb*)
2. An IMU9150 on the head, held by glasses (in short, *head*)
3. An ADXL345 [40] (±4G, 13-bit resolution) on the xiphoid process of the sternum (in short, *sternum*)
4. An IMU9150 on the left infraclavicular region, on the pectoralis major, just below the collarbone (in short, *infraclavicular region*)
5. A smartphone (Samsung Galaxy Trend Plus GT-S7580, accelerometer with ±2G, 12-bit resolution) on the left side of the lumbar inside a special bag (in short, *lumbar region*)

The sensors were selected for convenience, availability in our laboratory, and ease of use, with libraries for the Arduino platform available, although many commercial parts could have been selected too. They fulfill the requirements of the application (Section 2.2.1). Since a smartphone was used to gather data (as it would be in a real application of the system), we took advantage of its accelerometer to add another location (lumbar region). There was no relation between the sensor and location other than the convenience of placing the smartphone in the lumbar region with a commercial bag.

#### 2.2.1. Signal Acquisition

Signals were acquired through an Arduino Pro Mini [41], which gathered information from four sensors (inertial sensor) with a frequency of 50 Hz. The libraries of the inertial sensors were provided by SparkFun, making use of the I2C protocol for communication. The IMU9150 only has two addresses in the same I2C bus. Since our kit had three IMU9150, a second I2C bus was required. The Arduino Pro Mini has only one built-in I2C bus (hardware implementation). Therefore, the second one was emulated by software. All sensor information was sent in real time to a smartphone through an HC-05 Bluetooth interface [42] at a rate of 115200 bauds. This serial communication was the bottleneck in the transmission. Sensor data were transmitted as ASCII characters, and the maximum transmission frequency with that serial port speed was 68 Hz given the number of characters per sampling period in our experiment, higher than the selected frequency. While the smartphone collected information from the Arduino, it sensed meditator movement with its internal accelerometer at a frequency of 50 Hz.

The frequency was selected according to the following reasoning. Basically, the movement is expected to be under almost static conditions, thus a low-frequency measurement. Even normal movements like gait are of low frequency (gait is measured in 0.6–5 Hz in [43]). One could also consider the possibility of tremor induced by the posture. Enhanced physiological tremor and even pathological tremor are below 18 Hz [44], and other studies have reported detecting tremor caused by fatigue in a low frequency of 2–6 Hz in [45], or 2 Hz peak in [46]. Thus, a sampling frequency of 50 Hz was enough and allowed a comfortable wireless Bluetooth transmission. Minimal data losses were reported in our experiments, and the corresponding values were found by interpolation in the postprocessing. Data losses could be due to transmission errors or due to errors when storing data in files. Finally, experimentally, it seems that 50 Hz corresponds to one of the sampling options in the selected smartphone model (option “SENSOR_DELAY__GAME” in the Android source code [47]), with some variability since this device cannot deal with strict real time and the values have to be interpolated too.

After every meditation session, the information stored in a file was labeled with the name of the seat (chair, zafu, and meditation bench) and the subject's identifier.

#### 2.2.2. Signal Processing

The files were processed with MATLAB. The first 60 and last 30 seconds of each session were removed to avoid incorrect data coming from people sitting down or from the smartphone being removed from the special bag. Two types of processing were performed: First, the acceleration norm for each sensor was calculated, and then, its standard deviation (*σ*_a_) during each meditation session was extracted. This value is considered a conventional measure of stability that is sensitive to small and rapid changes in the posture, such as tremors, due to not being able to maintain the posture.

Since parts of different manufacturers were used, the processing was repeated by discarding the least significant bits of the most accurate sensors. In this way, we simulated a situation in which all the sensors had the same resolution (the worst one), and we tested the influence on the results.

**Figure 3:**
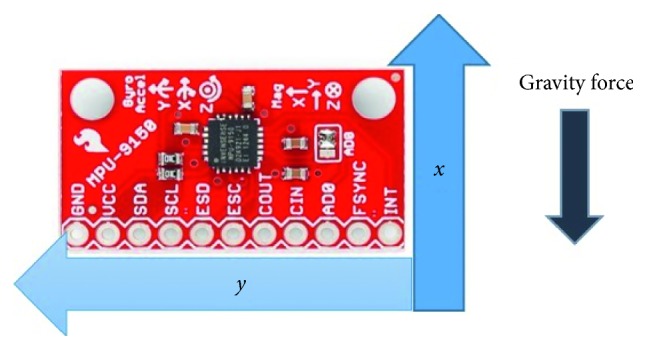
Approximate orientation of the sensor with respect to the Earth's gravity force.

Subsequently, the accelerometer signal was processed to obtain a global measure of changes in the volunteer's position. The idea was to distinguish major changes during the sessions. They could be caused by volunteers adopting a different position due to their inability to hold the previous one or due to slow posture variations. For this purpose, the study focused on the sensor placed on the head. Specifically, the sensor was placed on the temple of the glasses, since this location allows for an easy interpretation of deviations from the upright position in terms of acceleration components. The approximate orientation of the sensor is as shown in Figure 3. It is reasonable to assume that the acceleration is mainly due to gravity because the movements while meditating are slow. Besides, orientation values obtained from accelerometer readings were subjected to a low-pass filtering process (see (2)) to enhance their reliability. As shown in Figure 3, the main component of the acceleration is along the *x*-axis. Tilts around the left-right direction or around the anterior-posterior direction led to an increase of *y* and *z* components, respectively.

Thus, the angles of the acceleration with respect to the planes *xz* and *xy* were measured using the following equations:

(1) $$\begin{array}{c} \begin{array}{l} A_{xz}=\tan^{-1}\left(\frac{a_{y}}{\sqrt{a_{x}^{2}+a_{z}^{2}}}\right),\\ A_{xy}=\tan^{-1}\left(\frac{a_{z}}{\sqrt{a_{x}^{2}+a_{y}^{2}}}\right). \end{array} \end{array}$$

**Figure 4:**
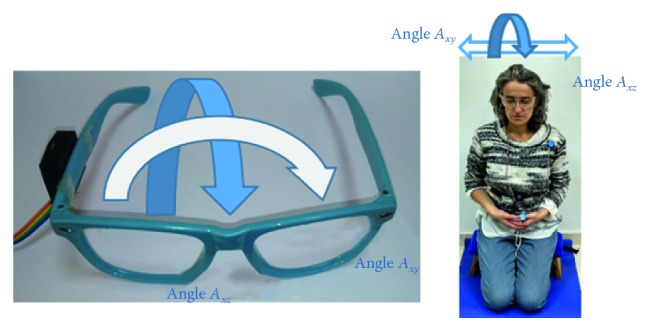
Direction of rotation angles with respect to glasses. Blue arrow = anterior-posterior (*A*_*xz*_); white arrow = left-right (*A*_*xy*_).

Figure 4 shows the two measured angles. The *A*_*xz*_ angle corresponds to an anterior-posterior movement, and the *A*_*xy*_ angle corresponds to a left-right movement.

**Figure 5:**
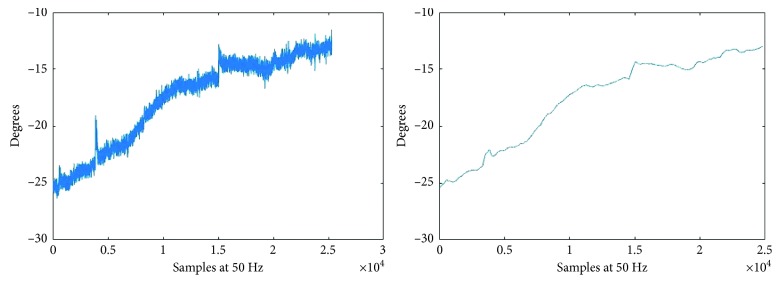
Signal before the filter (a) and signal after the filter (b).

After recording the angles, they were filtered using a moving average filter (500 samples; 10 s) to reduce noise ((2) and Figure 5), which effectively removed any dynamic component:

(2) $$\begin{array}{c} y\left(n\right)=\frac{1}{N}\sum_{i=0}^{i=N-1}x\left(n-i\right), \end{array}$$

where *N* = 500.

A global measure of the volunteer's change of position during the session was then obtained from the difference between the maximum and minimum values of the signal. For example, the signal in Figure 5 has a variation of 12.45°.

#### 2.2.3. Statistical Analysis

The standard deviation of the acceleration was analyzed using a two-way ANOVA with repeated measures on both factors, the sensor location and the seat (results in Section 3.1). Similarly, to analyze the global change of the posture (angle differences), an ANOVA with one factor (seat) was carried out, since, in this second case, only one sensor location was used (Section 3.2). The statistical analyses were performed with SPSS 12.0.

## 3. Results

### 3.1. Analysis of Accelerometer Standard Deviation

Two factors have been considered in the analysis: the meditation seat during the sessions (“seat”: chair, zafu, or meditation bench) and the location of sensors on the body (“sensor location”: thumb, head, sternum, infraclavicular region, or lumbar region).

Table 1 shows the results of an ANOVA with repeated measures on both factors. The details about the statistical tests performed are given in Appendix. The sensor location was found to be significant (*p* value < 0.05). On the other hand, neither the seat nor the interaction term was significant. Since the sensor location was found to be significant, we proceeded with a multiple comparison analysis to discover the root of the differences in terms of pairwise comparisons of sensor locations. The *p* values of the comparisons are shown in Table 2, after applying a standard Bonferroni's correction.

Table 3 shows the mean values obtained for each of the locations of the sensors, and Table 4 shows the mean values obtained for each of the seats (marginal values provided by SPSS). The marginal values are obtained by averaging over one of the factors, keeping only the other one. They can give a clue of the influence of a single factor. According to Tables 2 and 3, the lumbar position is the least sensitive since its differences with the others are significant and the mean value is the lowest one. Among the remaining positions (thumb, head, sternum, and infraclavicular region), the thumb shows the highest sensitivity, although the interdifferences are not significant, except with the sternum. The marginal values for the seat factor in Table 4 are presented for completeness and to show that the experimental values are very close.

The results of this statistical analysis remained almost the same after preprocessing the accelerometer to lower the resolution to the worst one as explained in Section 2.2.2. The only difference is that sternum and thumb did not differ significantly after that preprocessing.

### 3.2. Posture Variation Depending on the Seat

The results of the ANOVA for the anterior-posterior sway (*A*_*xz*_) and the left-right sway (*A*_*xy*_) are presented in Table 5.  Table 6 shows the values of the sway for the three seats.

The zafu seems to permit a better posture setting that can be held throughout the session since sways were lower. However, for *A*_*xz*_, the effect was not significant, while for *A*_*xy*_, it was. Therefore, a post hoc analysis was performed for *A*_*xy*_. The results are presented in Table 7. The pairwise comparisons indicate that the zafu seat was different from the others.

## 4. Discussion

Postural stability is an important factor during mindfulness meditation training. Inertial sensors can be a good option for monitoring posture dynamics due to their low cost and ease of use. One of the goals of this paper was to determine which is the most sensitive sensor location. Multiple protocols were described in the literature for the placement of wearable sensors on the human body for assessing, for instance, standing balance or walking stability. Most of them reported placing a wearable sensor on either the lumbar or sacral region of the trunk. To evaluate body posture stability during sitting meditation, Chang et al. [30] secured the measurement system between the abdomen and the chest or on the arm [31]. Manufacturers of commercial products chose other body positions: collarbone, head, lower back, and so on. The lumbar region is often selected since it is close to the body center of mass in a standing posture. However, the results of the present study indicate that the lumbar or sacral region is not the best option for measuring motion during sitting meditation since its *σ*_a_ value is the lowest and significantly different from the rest. It could be ruled out for future applications. Among the remaining locations, it is not possible to separate them into different groups and only the thumb is significantly different from the sternum. Therefore, convenience or comfort can be important factors to select the location. The head location requires a more bulky accessory. The infraclavicular region is less invasive, since the sensor is attached to clothes. The sternum and lumbar could be more accepted by users, since sensors attached to straps are common in commercial products for fitness. Finally, the thumb location would require a miniaturization of the system to improve comfortability. Usability, acceptance, and comfort should be evaluated among users. Although the differences are not statistically significant, the sensitivity of the head location was higher than the sensitivity of the sternum or the infraclavicular locations. This seems reasonable since the distance to the seat base is larger on the head. In fact, the order of the sensitivity among the locations head, infraclavicular region, sternum, and lumbar region followed the order of the distance to the seat. The thumb showed an even larger degree of movement, but this could be related to the movements of the hand that can be independent of upper body displacements. In fact, hand posture is very important according to traditional texts [48, 49], and in particular, thumb fingers should not drop or rise but remain in a perfect horizontal position [50].

The other goal of this paper was to analyze the influence of the seat to recommend a specific one based on data obtained using inertial sensors. In the oriental tradition, meditation is performed while seated on a cushion, preferentially in the full-lotus or half-lotus position. The static position is not to be changed until the end of the meditation time. Western teachers and mindfulness professionals are not so strict about this point: instead of a cushion (zafu), a comfortable chair or a meditation bench can be used too. A priori, one could think that the chair should give some advantage, since most of the volunteers had no meditation experience and, therefore, they were not used to sit on the zafu or the meditation bench, which were probably unknown for them. The only significant difference was related to the global change in posture throughout the session (left-right sway). The zafu provided the best seat with significant differences from the chair and the meditation bench, in keeping with the oriental tradition. However, anterior-posterior sway and *σ*_a_ were not significantly different. Thus, it might seem reasonable to advise using the zafu. This is consistent with the research in Zen sitting postures. The use of the zafu to maintain fixed postures has been applied in other fields such as in the development of a chair for microscopic surgery [51] or in the improvement of the sitting posture for children in the classrooms [52].

However, other aspects such as comfort should be considered by western and beginner meditators. In this regard, some people reported pain in the anterior part of the ankle when sitting on a meditation bench. People with back pain or difficulty getting up from a low seated position should consider a comfortable chair [17].

In the context of breathing interventions, this study has served to give a tip on the seat for meditation used in the beginning stages of training. It is supported by stability measures. The determination of sensor location is important to reduce the number of locations to be monitored in view of future studies, in which the relation between stability and beneficial outcomes of mindfulness should be investigated. Anyway, the results of this study are preliminary and should be taken with caution.

One of the limitations of this work is the number of volunteers, which corresponds to a pilot study to check the wearable sensor kit. The location of the sensors also entails some random components due to difficulties placing them in an exact position. However, these differences are very low compared to the distance between different locations. Another concern is the fact that the accelerometers used in this study include parts from different manufacturers. However, the measurements were taken in almost static situations, and therefore, their range was irrelevant. Besides, we performed the analysis described in Section 2.2.2 to lower the resolution of the sensors to a common baseline. The main conclusions of the statistical analyses remained the same after this preprocessing.

## 5. Conclusions and Future Lines of Work

Several review articles support the usefulness and effectiveness of mindfulness techniques in health and well-being. Thus, many people are starting to practice mindfulness. In the beginning stages, daily practice between 5 and 15 minutes is recommended, in which the stable posture is a key component. In this work, we have presented a set of inertial sensors to measure the motion and change in posture. The conventionally used lumbar region is not the best body location for sensors since it presents lower sensitivity. Besides, out of all the meditation seats, the zafu has some advantages in one out of three parameters obtained in this study, concerning the overall change in posture from the left-right sway.

In summary, a kit for measuring stability in meditation could consider a single sensor placed at the head, if the user is willing to wear an accessory like a pair of glasses, or attached to clothes at the infraclavicular region. An additional sensor at the thumb finger could also be used in certain forms of meditation [50] since its movements are considered by many meditation masters as a direct indication of the meditation depth. In addition, beginners could start practicing meditation in chairs or meditation benches, although a transition to the zafu is recommended for a medium term.

The present study could be improved in several ways. Further improvements in the orientation angles can be obtained with more complex low-pass filters or estimation with data fusion of the accelerometer, gyroscope, and magnetometer using compensatory filters. Thus, gravity could be determined more properly. With all these, the study could go beyond the assumptions of a single force in the system (gravity) or a fixed orientation of the glasses sensor. Another hardware issue is power consumption, especially if longer sessions were to be recorded. It should be measured and reduced, for instance, using low-power Bluetooth (for instance, the module CC2541). The possibility of data losses in the Bluetooth transmission should also be considered and handled in a future smartphone application. The measurements should be extended with more volunteers, improving this pilot research. In a future study, the sensors will be used to provide feedback information. The effect of this feedback on mindfulness training programs and self-regulation will be explored. Specifically, changes in the proprioception (the ability to sense the position, location, and movement of the body and its parts) could be evaluated with the practice. Moreover, the role of movement and posture in bringing about beneficial outcomes of mindfulness-based interventions should be investigated.

## Figures and Tables

**Table 1:** ANOVA results for *σ*_a_ (*p* values).

Factor	*p* value
Seat	0.744
Sensor location	**0.000**
Seat ∗ sensor location	0.726

*p* values less than 0.05 are in bold.

**Table 2:** Sensor location analysis: pairwise comparisons with Bonferroni's correction.

	Thumb	Head	Sternum	Infraclavicular region
Head	1.000	—	—	—
Sternum	**0.022**	0.329	—	—
Infraclavicular region	0.787	1.000	1.000	—
Lumbar region	**0.000**	**0.000**	**0.000**	**0.000**

*p* values less than 0.05 are highlighted in bold.

**Table 3:** Sensor location marginal mean values of *σ*_a_ (in g).

Sensor location	Mean
(1) Thumb	0.0083
(2) Head	0.0068
(3) Sternum	0.0054
(4) Infraclavicular region	0.0060
(5) Lumbar region	0.0025

**Table 4:** Seat marginal mean values of *σ*_a_ (in g).

Seat	Mean
(1) Zafu	0.0059
(2) Chair	0.0056
(3) Bench	0.0060

**Table 5:** ANOVA results for the anterior-posterior sway (*A*_*xz*_) and the left-right sway (*A*_*xy*_) versus the seat.

	*A*_*xz*_ versus seat	*A*_*xy*_ versus seat
*p* value	0.196	**0.002**

*p* values less than 0.05 are highlighted in bold.

**Table 6:** Values obtained for each seat considering anterior-posterior and left-right sways (*A*_*xz*_ and *A*_*xy*_ in degrees).

Seat	*A*_*xz*_	*A*_*xy*_
Zafu	6.78 (7.51)	5.83 (5.11)
Chair	8.48 (8.62)	10.01 (9.72)
Meditation bench	10.13 (12.92)	9.18 (6.87)

Values are given as mean (SD).

**Table 7:** Seat analysis of the left-right sway (*A*_*xy*_): pairwise comparisons with Bonferroni's correction.

	Zafu	Chair
Chair	**0.019**	—
Meditation bench	**0.006**	1.000

*p* values less than 0.05 are highlighted in bold.

**Table 8:** ANOVA results for *σ*_a_ (*p* values).

Mauchly's test of sphericity	Seat	0.239
	Sensor location	**0.000**
	Seat ∗ sensor location	**0.000**
Pillai's trace	Seat	0.675
	Sensor location	**0.000**
	Seat ∗ sensor location	0 726
Sphericity assumed	Seat	0.744
	Sensor location	**0.000**
	Seat ∗ sensor location	0.725

**Table 9:** ANOVA results for the sway (*A*_*xz*_ and *A*_*xy*_ versus seat) (*p* value).

	*A*_*xz*_	*A*_*xy*_
Mauchly's test of sphericity	**0.000**	**0.037**
Pillai's trace	0.196	**0.002**
Sphericity assumed	0.299	**0.006**

## Data Availability

The data used in this article are available upon request from the first author.

## Appendix

### Detailed Description of the Statistical Test

In this section, a more detailed description of the statistical tests is given. For the ANOVA analysis of *σ*_a_ with repeated measures (factors: sensor location and seat), the results are shown in Table 8. Neither the sensor location factor nor the interaction factor fulfilled the sphericity condition so that for them, the *p* value of Pillai's trace was selected. Other common statistics provided by SPSS like the Greenhouse–Geisser correction led to the same conclusion as Pillai's trace.

The ANOVA results of the analysis of the sway (*A*_*xy*_ and *A*_*xz*_) with respect to the seat are shown in Table 9. No measurement fulfilled the sphericity test, so the value of Pillai's trace was selected. In the case of *A*_*xy*_, the null hypothesis was rejected. Other common statistics of SPSS (Greenhouse–Geisser correction) led to the same conclusion.
